# Do Gadolinium‐Based Contrasts Represent a High Risk for Genotoxicity in Mammalian Cells? A Systematic Review

**DOI:** 10.1002/jat.4814

**Published:** 2025-05-28

**Authors:** Thiago Guedes Pinto, Rogerio Aparecido Dedivits, Daniel Araki Ribeiro

**Affiliations:** ^1^ Department of Biosciences, Institute of Health and Society Federal University of São Paulo (UNIFESP) Santos São Paulo Brazil; ^2^ Department of Head and Neck Surgery, School of Medicine University of Sao Paulo (USP) São Paulo São Paulo Brazil

**Keywords:** DNA damage, gadolinium, genotoxicity, mammalian cells, risk assessment

## Abstract

The scientific rationale for this review stems from the increasing global use of gadolinium‐based contrast agents in medical imaging and the concerns over the long‐term environmental accumulation of gadolinium waste, which may pose biological risks. The primary objective was to determine whether gadolinium exposure induces genetic damage in mammalian cells, regardless of the assay method used, and to assess the quality of the studies available in the literature. Genotoxicity was measured through assays such as the micronucleus test, comet assay, chromosomal aberration, and sister chromatid exchange. A total of 17 studies were included being 11 studies (out of 17) with positive genotoxic effects, suggesting that gadolinium can induce DNA damage. Most of the studies (12 out of 17) were rated as “strong” or “moderate” in quality, providing reliable evidence for these findings. This review advances the current understanding of gadolinium's potential health risks by highlighting its genotoxic effects.

## Abstract 2

This study evaluated whether gadolinium exposure induces genetic damage in mammalian cells. The approach provides evidence suggesting that gadolinium exposure may induce genotoxicity, particularly under high‐dose or prolonged exposure scenarios. In this sense, we recommend that magnetic resonance imaging scans with gadolinium contrast should be prescribed only when absolutely necessary.

## 1 Introduction

In humans, veterinary, and dental medicine, various diagnostic exams are commonly used to aid clinicians in diagnosing medical conditions and preparing treatment plans. Among these, medical imaging plays a pivotal role in providing precise visual representations of anatomical and physiological structures, such as tissues and organs, facilitating accurate diagnoses and treatment decisions (Laal 2013).

However, when focusing on gadolinium and its application, it is crucial to delve into the specifics of its use and the associated risks. Gadolinium, a rare‐earth metal with the atomic number 64, is widely used as a contrast agent in magnetic resonance imaging (MRI) due to its paramagnetic properties. This element significantly enhances image resolution, particularly in cases where detailed tissue differentiation is necessary. While MRI is generally considered a safe, nonionizing radiation technique, the widespread use of gadolinium raises concerns about its potential long‐term effects, especially regarding its retention in tissues and its environmental impact after use (Cho et al. 2014; Perazella 2009).

In the context of gadolinium's application, there is a growing need for assessing its genotoxicity. Genotoxicity testing is essential to evaluate the potential damage gadolinium might cause to genetic material within living organisms. These tests not only help understand the direct risks associated with gadolinium exposure but also ensure the safety of medical practices involving this contrast agent. Recent studies emphasize the importance of genotoxicity testing for substances commonly used in medical imaging, including gadolinium‐based contrast agents (GBCAs), which can accumulate in tissues and potentially induce genetic damage (Cobanoglu 2022). Among the most common assays used for genotoxicity screening are the micronucleus and comet assays, which are inexpensive, straightforward to perform, and have widespread global use. These assays have been validated as sensitive methods for detecting DNA damage in mammalian cells exposed to environmental and medical agents (Araldi et al. 2015). Additionally, tests like sister chromatid exchange (SCE) and chromosomal aberration (CA) can also be implemented to provide a broader understanding of the substance's genetic impact (Guedes Pinto et al. 2024; Bonassi et al. 2011). It is important to stress that each genotoxicity test described above assesses the effects on genetic material from a different perspective. While the comet assay detects DNA damage by single‐strand breaks, double‐strand breaks, DNA adducts, and incomplete alkaline sites, the micronucleus, SCE, and CA tests assess mutations induced by chromosome breaks/loss or even mitotic spindle disruption (clastogenic or aneugenic agents). Taken as a whole, the combination of some genotoxicity tests in the evaluation of chemical agents is timely, contributing not only to the correct interpretation of the results but also to the evaluating the risk assessment including their impact on the initiation phase of chemical carcinogenesis (Menz et al. 2023).

Although gadolinium's toxicity, particularly nephrotoxic and neurotoxic effects have been extensively studied, the potential genotoxicity of gadolinium remains underexplored, particularly in mammalian cells (Akbas et al. 2023). To the best of our knowledge, there is no study that evaluated the genotoxicity induced by gadolinium‐based contrasts in mammalian cells by means of systemic review of the literature. Therefore, this review builds upon it, providing a comprehensive, systematic analysis of the genotoxic risks of gadolinium exposure, aiming to clarify existing discrepancies and offer more reliable conclusions. This systematic review aims to evaluate both the genotoxic effects of gadolinium and the quality of the research to date, offering a clearer understanding of the risks involved.

## 2 Material and Methods

### 2.1 Eligibility Criteria

This systematic review followed the guidelines outlined in the Preferred Reporting Items for Systematic Reviews and Meta‐Analyses (PRISMA) 2020 statement. The PICO framework used in this review was as follows: Population (mammalian cells), Intervention (gadolinium), Comparison (control group), and Outcome (genotoxicity).

Studies were deemed eligible for inclusion if they satisfied the following conditions: (1) They investigated genetic damage, (2) they were published in English, and (3) they followed rigorous scientific standards in the presentation of data. Studies were excluded if they met any of the following criteria: (1) conference abstracts, reviews, commentaries, editorials, or letters to the editor; (2) full‐text unavailable or not in English; (3) inability to access or extract relevant data; (4) studies that combined gadolinium exposure with other genotoxic agents (physical, chemical, or biological); (5) studies involving multigenerational effects; (6) research that did not evaluate genotoxicity; and (7) studies presenting incomplete or unclear results.

### 2.2 Data Search

We performed searches in PubMed, SCOPUS, and Web of Science to identify relevant articles as of May 2024 using the following keywords and Boolean operators: (“Gadolinium”) AND (“Genotoxicity” OR “genetic damage” OR “DNA breakage” OR “DNA damage” OR “DNA injury” OR “chromosome damage” OR “genetic injury”) AND (“Sister chromatid exchange”) OR (“Micronucleus assay”) OR (“Chromosomal aberration test”). Additionally, a manual search of references and cited/related articles was carried out. Search terms were validated to ensure they retrieved a representative selection of relevant literature. Searches were limited to articles published in English with no restrictions on publication date. Abstracts were independently reviewed by two assessors (T.G.P. and D.A.R.), and full‐text evaluations were performed to confirm eligibility. Discrepancies between reviewers were resolved through discussion, achieving consensus. The data strategy is outlined in Table 1.

**TABLE 1:** Electronic databases utilized and search strategy.

Electronic databases utilized	Search strategy (May 2024)
PubMed https://www.ncbi.nlm.nih.gov/pubmed/Scopus Scopus https://www.scopus.com	(Gadolinium) AND (DNA Damages) OR (Damage, DNA) OR (Damages, DNA) OR (DNA Injury) OR (Injury, DNA) OR (DNA Injuries) OR (Injuries, DNA) OR (Genotoxic Stress) OR (Stresses, Genotoxic) OR (Genotoxic Stresses) OR (Mutagenicity) OR (Genotoxicity) OR (Comet assay) OR (Micronucleus assay) OR (Stresses, Genotoxic) OR (micronucleus) OR (micronucleated cell) OR (chromosome damage) OR (chromosomal injury) OR (chromosome breakage) OR (chromosome aberration test) OR (sister chromatid exchange test) OR (chromosome aberration test).
Web of Science https://www.webofscience.com/wos/alldb/basic‐search	

### 2.3 Data Extraction and Study Quality Evaluation

Data extraction was carried out independently by two reviewers (T.G.P. and D.A.R.). The extracted information included: authors, year of publication, cell types used in studies, exposure duration, methods for genotoxicity assessment, number of cells analyzed, statistical techniques employed, and key results. Any disagreements between the reviewers were resolved through discussion, but no divergences between the reviewers on the identification of confounders occurred.

To assess the quality of the studies, a detailed evaluation was performed considering the presence of confounding variables. Confounders, as defined by Malacarne et al. (2022), are external factors that might distort the relationship between gadolinium exposure and genotoxicity outcomes. Studies were rated based on their ability to control for these variables. Articles that controlled for all identified confounders were classified as STRONG, those that controlled for one confounder were rated as MODERATE, and studies that did not control for multiple confounders were classified as WEAK. This approach was inspired by the framework set out by Guedes Pinto et al. (2024). To clarify the rating strategy, each confounder was considered equally important, and studies were rated based on the number of confounders controlled for.

## 3 Results

### 3.1 Study Selection

**FIGURE 1:**
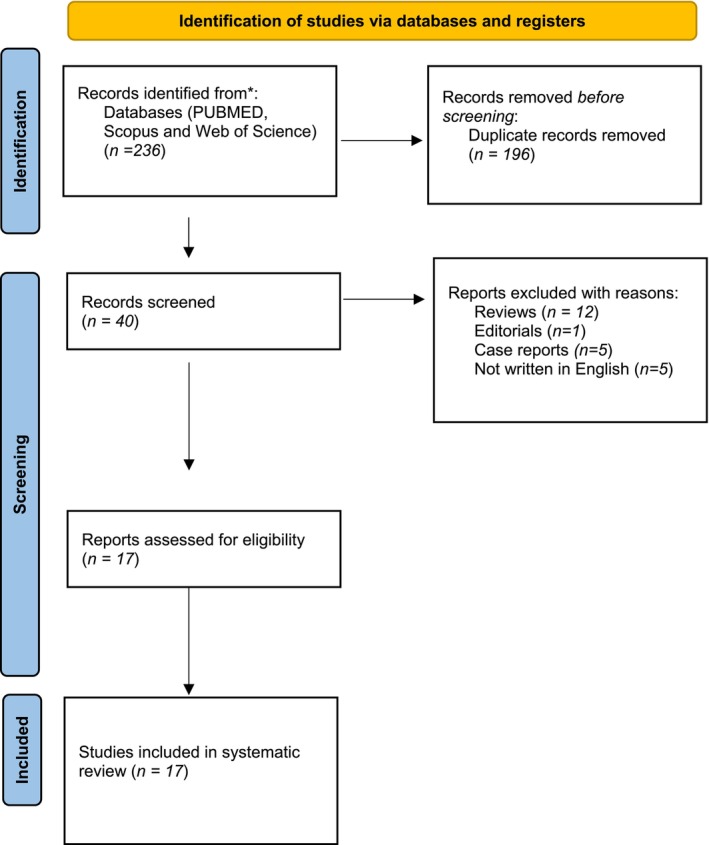
Flow chart of the study.

The preliminary online data search uncovered 236 scientific records, of which 196 were duplicates and, consequently, removed. Following an assessment of titles and abstracts, 23 studies were deemed irrelevant to the study's objectives and were discarded because they were reviews, case reports, commentaries, editorials, non‐English papers, or letters to the editor. The authors of this article thoroughly reviewed the full manuscripts of 17 studies. The flow chart of this study is presented in Figure 1.

### 3.2 General Characteristics of the Included Studies

The key features of the assessed studies are displayed in Table 2. As for the origin of the included studies, four were conducted in Germany, three in the United States, two in China, three in Turkey, and one in each of the following countries: Japan, Singapore, Korea, Malaysia, and India. The publication years of the articles included in this study varied from 1993 to 2022. Additionally, all the included studies addressed the use or exposure to gadolinium. Such data are presented in Table 2.

**TABLE 2:** The most important characteristics of the studies included in the systematic review.

Author	Year of publication	Country
Akbas et al.	2022	Turkey
Cobanoglu	2022	Turkey
Siew et al.	2020	Malaysia
Friebe et al.	2018	Germany
Alarifi et al.	2017	India
Cho et al.	2014	Korea
Avti et al.	2013	USA
Setyawati et al.	2013	Singapore
Wack et al.	2012	Germany
Yildiz et al.	2011	Turkey
Feng et al.	2010	China
Döhr et al.	2007	Germany
Steger‐Hartmann	2006	Germany
Donnelly et al.	2005	USA
Wible et al.	2001	USA
Yongxing et al.	2000	China
Yamazaki et al.	1993	Japan

### 3.3 Variables Related to Gadolinium Use and Exposure and Genotoxicity

Table 3 outlines the variables associated with gadolinium and genotoxicity. All studies included negative control groups for accurate comparison.

**TABLE 3:** Variables analyzed in the in vitro studies in chronological order of publications.

Author	Cell type	Study nature	Assays	No. of evaluated units	Staining method	Evaluated parameters	Inclusion criteria	Gd concentration	Additional exposure	Cytotoxicity analysis	Blind analysis	Proper statistical description	Control/baseline
Akbas et al.	Peripheral blood lymphocytes (3 donors)	In vitro	MN	3000	Giemsa	Cell count	Yes	Gadobutrol (875, 1750, 3500, 7000, 14,000, 28,000, 56,000, 112,000, 604,000 μg/mL) Gadoversetamide (1750, 3500, 7000, 14,000, 28,000, 56,000, 112,000, and 330,000 μg/mL)	—	Yes: Mitotic index	No	Yes	Yes
			Comet	300		Tail moment and tail intensity							
			SCE	100		SCE frequency							
Cobanoglu	Peripheral blood lymphocytes (2 donors)	In vitro	MN	4000	5% Giemsa	Cell count	Yes	0.25, 1.0, 2.5, and 5.0 mM (for 48 h)	—	Yes: Cytokinesis‐blocked proliferation index	No	Yes	Yes
Siew et al.	Chinese hamster lung V79‐4 fibroblast cells	In vitro	Comet	50	Ethidium bromide	Tail intensity and tail moment	Yes	0.25, 0.5, and 1.0 mM	—	Yes: MTT	No	Yes	Yes
			MN	N/A (comparison of frequency)	Acridine orange	Frequency of formed MN (chart plotting against concentration or control)							
Friebe et al.	Lymphocytes (human donors—male and female—exposed: 13; control: 16)	In vitro (donor with mean age 35.8)	DNA double‐strand breaks	370	γH2AX staining	γH2AX foci	Yes	2 mM (simulation of 0.1‐mmol GBCA per kilogram) 20 nM	MRI	Yes: Apoptosis detection (cell viability)	No	Yes	Yes
Alarifi et al.	Human neuronal cells (SH‐SY5Y)	In vitro	Comet assay	100	Hoechst 33258 Ethidium bromide Rhodamine‐123	Tail intensity and tail moment	Yes	0, 10, 25, 50, and 100 μg/mL	—	Yes: MTT	No	Yes	Yes
			RT‐PCR			Genotype frequencies of Bcl‐2 and Bax							
Cho et al.	Lymphocytes (1 female human donor)	In vitro	MN	1000	Giemsa	Cell count	Yes	0, 0.2, 0.4, 0.8, or 1.2 mM	ELF‐EMF	Yes: Cell viability (trypan blue dye exclusion)	Yes	Yes	Yes
			Comet			Tail intensity and tail moment							
Avti et al.	NIH/3T3 fibroblasts (mouse)	In vitro	MN	—	Acridine orange	Cell count	Yes	0.1–100 μg/mL (various concentrations)	MRI	Yes: Cell viability (trypan blue dye exclusion)	No	Yes	Yes
Setyawati et al.	Human neonatal foreskin fibroblast cells	In vitro	γH2AX expression	150	γH2AX staining and DAPI	Cell count	Yes	300 and 1000 μg/mL	—	Yes: Cell proliferation assay	Yes	Yes	Yes
Wack et al.	Chinese hamster cells V79, Human peripheral blood Lymphocytes and *Salmonella typhimurium* and *Escherichia coli*	In vitro and in vivo	MN	2000	Giemsa	Cell count	Yes	0.1–1.0 mmol Gd/mL (various concentrations)	S9‐mix	Yes: Local tolerance test	No	Yes	Yes
Yildiz et al.	Human peripheral blood Lymphocytes	In vivo	Comet	100	Ethidium bromide	DNA fragmentation	Yes	—	—	No	Yes	Yes	Yes
Feng et al.	Rat cortical neurons	In vitro	DNA fragmentation assay	—	Ethidium bromide	DNA fragmentation	Yes	2, 20, and 100 mM	—	Yes: Lactate dehydrogenase release assay	Yes	Yes	Yes
Döhr et al.	Han mouse: NMRI (females and males) Han rat: Wistar (males) Newly weaned Han rat: Wistar (males) Beagle dog (one male and two females)	In vivo	MN	—	May–Grünwald and Giemsa	Cell count	Yes	Formulation A (500 mM) and Formulation B (250 mM) of Gd‐EOB‐DTPA	Cyclophosphamide and *N*,*N*‐dimethylhydrazine dihydrochloride	No	No	Yes	Yes
Steger‐Hartmann	Mouse bone marrow cells	In vivo	MN	—	May–Grünwald and Giemsa	Cell count	Yes	1000, 2000, and 4000 mg/kg	—	No	No	Yes	Yes
Donnelly et al.	EMT6 mouse mammary tumor cells	In vitro	Comet	100	SYBR‐Green	Tail moment	Yes	25, 50, 75, and 100 μgM	Radiation	Yes: Clonogenic assay	No	Yes	Yes
Wible et al.	Mouse bone marrow cells	In vivo	MN	—	—	Cell count	Yes	1250, 2500, and 5000 mg/kg	Cyclophosphamide	No	No	Yes	Yes
Yongxing et al.	Peripheral blood lymphocytes (1 male human donor)	In vitro	MN	2000	—	Cell count	Yes	0.016, 0.040, 0.100, 0.250, and 0.625 mM	—	Yes: 24‐h acute assay	Yes	Yes	Yes
			SSB	—	—	Single‐strand break index							
			UDS	—	—	Unscheduled DNA synthesis index							
Yamazaki et al.	Peripheral blood lymphocytes	In vitro	SCE	—	Giemsa	SCE frequency	Yes	1.25 (0.2 mL/kg), 12.2, and 100 mM	MRI	No	No	Yes	Yes

*Note:* The micronucleus count was based on the incidence of these events within a number of cells counted (micronuclei frequency).

Abbreviations: — = not described; γH2AX = gamma H2AX (a phosphorylated form of the histone protein H2AX); C = control group; CA = chromosome aberrations; Gd = gadolinium; kg = kilogram; mg = milligram; mL = milliliter; mM = millimolar; mmol = millimole; MN = micronucleus assay; MTT = 3‐(4,5‐dimethylthiazol‐2‐yl)‐5‐(3‐carboxymethoxyphenyl)‐2‐(4‐sulfophenyl)‐2*H*‐tetrazolium assay; N/A = not applicable; nM = nanomolar; SCE = sister chromatid exchange; SSB = single‐strand break; SYBR = synthetic yellow binding reagent; UDS = unscheduled DNA synthesis.

While 10 studies (out of 17) conducted the micronucleus assay, 5 (out of 17) conducted the comet assay, and 2 conducted the SCE assay. Also, three studies conducted the following studies each, respectively, DNA double‐strand breaks assays, γH2AX expression assay, and DNA fragmentation assay. The final number of studies does not add up to 17 as some of them performed more than one assay in the same study. Such findings can be seen in Table 3.

Concerning the studies that conducted the micronucleus assay, five (out of 10) evaluated lymphocytes and five evaluated other sites/cells, such as fibroblasts (Chinese hamster lung V79‐4 and NIH/3T3) and bone marrow cells. Regarding the studies that carried out the comet assay, the evaluated cells/sites were as follows: Chinese hamster lung V79‐4 fibroblast cells, human neuronal cells (SH‐SY5Y), human lymphocytes, and EMT6 mouse mammary tumor cells. As for the studies that conducted other tests, different types of cells were used namely human neonatal foreskin fibroblast cells, rat cortical neurons, and human lymphocytes.

Also, out of the 17 included studies, all of them included a control or baseline group in the analysis. Concerning the use of blind analysis in the assessment, only five (out of 17) reported having carried it out throughout the manuscripts. Such findings can also be seen in Table 3.

As for the number of evaluated cells, there has been a variation among the studies. Nonetheless, considering all studies that performed the micronucleus assay, only five clearly reported having analyzed at least 1000 cells. As for the ones that performed the comet assay, all of them reported to have analyzed at least 50 cells. Such results are consolidated in Table 3.

### 3.4 Main Results

A total of 11 (out of 17) of the evaluated studies presented increased genotoxicity because of gadolinium exposure in at least one of the investigated cell types, based on at least one genotoxic assay, as shown in Table 4. Curiously, the majority of the studies that reported genotoxicity cleared mentioned a dose–response relationship, suggesting longer gadolinium exposure could have a more deleterious impact on the studied cells.

**TABLE 4:** Main findings evaluating the genotoxicity induced by gadolinium in mammalian cells.

Authors	Genotoxicity	Dose response	Exposure measurement	Other findings
Akbas et al.	↑ CAs in human lymphocytes (gadobutrol and gadoversetamide) ↑ Tail intensity in comet assay ↑ SCE ↑ DNA damage (comet assay, tail length, and % tail DNA)	Yes	24 and 48 h	Increased genotoxicity observed from 7000 μg/mL onwards
Cobanoglu	↑ MN in human peripheral blood (gadoversetamide)	Yes	48 h	↓ CBPI
Siew et al.	↑ MN in Chinese hamster lung V79‐4 fibroblast cells	Yes	3 h at 24 h	—
Friebe et al.	No statistical difference	No	1 h	—
Alarifi et al.	↑ DNA fragmentation in mouse fibroblasts	Yes		↑ Apoptosis rate
				↓ MMP
Cho et al.	↑ MN in human lymphocytes ↑ Single‐strand DNA breaks in human peripheral blood	Yes	44 h	↑ Apoptosis rate
				↑ ROS production
Avti et al.	No statistical difference	No		↑ Apoptosis rate
Setyawati et al.	↑ DNA damage (γH2AX expression.) in mouse fibroblasts	Yes		—
Wack et al.	No statistical difference	No	4 weeks	No evidence of possible contact allergenic or immunotoxic effects
Yildiz et al.	↑ DNA damage (comet assay) in lymphocytes	No		Serum visfatin levels were statistically significantly increased in samples withdrawn after contrast
Feng et al.	↑ DNA fragmentation in rat cortical neurons	Yes		↑ Apoptosis rate
Döhr et al.	No statistical difference (Gd‐EOB‐DTPA)			No indications of reproductive and developmental toxicity, potential contact allergenic
Steger‐Hartmann		Yes	6 days at 4 weeks	↑ ROS production
Donnelly et al.	↑ Single‐strand DNA breaks in EMT6 mouse mammary tumor cells (at the highest dose)	Yes		↑ ROS production
Wible et al.	No statistical difference	Yes	24, 48, and 72 h	—
Yongxing et al.	↑ MN in human peripheral blood at the 0.25 and 0.625 doses	Yes	48 h	↓ DNA repair
Yamazaki et al.	↑ SCE	Yes	1st approach: 69 h	1st approach: Blood culture with Gd‐DTPA for 69 h showed an increase.
			2nd approach: 1 and 3 h (higher increase than 1 h)	2nd approach: 1 and 3 h exposure to Gd‐DTPA followed by washing and lymphocyte culture also showed an increase.

Abbreviations: ↑ = increase; ↓ = decrease; γH2AX = gamma H2AX (a phosphorylated form of the histone protein H2AX); CA = chromosome aberration; DNA = deoxyribonucleic acid; EMT = epithelial–mesenchymal transition; h = hour(s); MMP = mitochondrial membrane potential; MN = micronucleus; SCE = sister chromatid exchange.

### 3.5 Quality Assessment

The quality assessment is detailed in Table 5. Among the 17 studies, four were rated as strong, eight as moderate, and five as weak, resulting in a total of 12 studies classified as either Strong or Moderate. It is noteworthy that, in our analysis, the following confounders were included, as follows: blind analysis, appropriate statistical description, staining method, metanuclear assessment, amount of cells evaluated, and inclusion criteria.

**TABLE 5:** Quality assessment (final rating of the studies according to confounders analysis).

Author	No. of confounders (uncontrolled variables)	Uncontrolled variable (confounder)	Rating
Akbas et al.	1	No blind analysis	Moderate
Cobanoglu	1	No blind analysis	Moderate
Siew et al.	1	No blind analysis	Moderate
Friebe et al.	1	No blind analysis	Moderate
Alarifi et al.	1	No blind analysis	Moderate
Cho et al.	0	—	Strong
Avti et al.	2	No blind analysis and no data on cell amount	Weak
Setyawati et al.	0	—	Strong
Wack et al.	1	No blind analysis	Moderate
Yildiz et al.	2	No cytotoxicity test and no control group	Weak
Feng et al.	0	—	Strong
Döhr et al.	2	No blind analysis and no cytotoxicity test	Weak
Steger‐Hartmann	2	No blind analysis and no cytotoxicity test	Weak
Donnelly et al.	1	No blind analysis	Moderate
Wible et al.	2	No blind analysis and no cytotoxicity test	Weak
Yongxing et al.	0	—	Strong
Yamazaki et al.	1	No blind analysis	Moderate

*Note:* The final rating considered the number of uncontrolled variables, and only studies that managed to control all variables were classified as strong.

## 4 Discussion

Although MRI is considered relatively safe in medical and dental practice, potential health risks related to this imaging exam have been discussed over the last few years, and according to some studies, the use of contrast agent may be one of the factors related to previously observed DNA damage (Simi et al. 2008). For this reason, the aim of this systematic review was to evaluate whether gadolinium exposure induces genotoxicity in mammalian cells, considering its widespread use in medical imaging procedures. The increasing reliance on GBCAs highlights the need to thoroughly understand their safety profiles, particularly regarding genetic damage.

The chosen method can accurately pinpoint the effects of genotoxic agents on specific tissues, evaluating the repercussions of exposure to potentially dangerous chemical substances. In this context, it is noteworthy that some authors hypothesize that the mechanism of genotoxicity related to patient exposure to MRI may be owing to the interaction between gadolinium and the application of an electromagnetic field, rather than simply the exposure to gadolinium itself (Yildiz et al. 2011). In this context, this study included both studies with the combination of MRI and gadolinium and GBCAs and studies that did not have exposure to MRI.

Furthermore, the studies included in this review vary in quality, with four rated as strong, eight as moderate, and five as weak. This variation suggests that while there is a general trend towards identifying genotoxicity, the reliability of some findings may be compromised by methodological limitations. For example, the absence of blind analysis in many studies may have introduced bias, potentially affecting the conclusions. Anyway, the majority of the studies (12 out of 17) evaluated were rated as “strong” or “moderate” in quality, providing reliable evidence for these findings.

Regarding the methodology, as seen in the results, some studies conducted the micronucleus assay, but not all of them followed the best practices necessary to ensure the high quality and reliability of the results. In this sense, when a study did not meet a criterion recommended by the Micronucleus Assay Expert Group, its score was negatively impacted. Among the considered interference factors, we examined, for instance, whether the studies performed a cytotoxicity test and whether they evaluated the minimum required number of cells (1000 for lymphocytes and 2000 for oral mucosa cells) (Bonassi et al. 2009). In this case, all studies that conducted the micronucleus assay adhered to both the cytotoxicity test and the minimum required number of analyzed cells. Nonetheless, a confounding factor that was commonly observed was the failure to use blind analysis, which could lead to biased conclusions.

Under the same rationale that the use of proper parameters is mandatory to achieve high quality in research, we also evaluated whether studies properly conducted the comet assay. Such an analysis was based on certain set of information, such as the minimum required number of evaluated cells (50) and the usage of tail intensity as DNA damage parameter (golden standard) (Hartmann et al. 2003; Speit and Hartmann 1995). Similar to the studies that performed the micronucleus assay, all studies that conducted the comet assay followed the recommendations on the minimum number of evaluated cells, but many of them also failed to either conduct or clearly report the analysis in a blinded manner.

In this sense, it is coherent to state that genotoxicity plays a role in the development of several chronic degenerative diseases, such as neoplasia. This phenomenon may be a leading factor in the onset of illnesses, especially by means of mechanisms that entail DNA strand breaks and/or chromosome damage (Maluf and Erdtmann 2000). In this case, 11 studies detected DNA injury induced by gadolinium through a genotoxicity assay, which raises a flag concerning the safety of gadolinium exposure. As for the question that motivated this study from the very beginning, it is important to highlight that our concern was satisfactorily answered according to our standards, that is, most articles converged on the finding that gadolinium exposure may indeed induce DNA damage, suggesting, therefore, a potential genotoxic risk for patients who make use of this resource for diagnosis purposes. Herein, it is important to explore the underlying genotoxic mechanisms that are influenced by the chemical structure of these agents. In fact, the structural characteristics of GBCAs, including their ionic/nonionic and macrocyclic/linear nature, are crucial factors that influence their kinetic stability and, ultimately, their genotoxic profiles. Ionic GBCAs tend to be more stable but can cause greater toxicity due to the release of free gadolinium ions in the body, whereas nonionic agents are generally considered less toxic. Additionally, macrocyclic GBCAs, which are more stable due to their closed‐ring structure, are often preferred for clinical use as they exhibit lower toxicity compared to linear agents (Iyad et al. 2023). Following the same rationale, gadobutrol has been shown to exert cytogenotoxic effects, and these effects are likely related to the molecular interactions between gadolinium and cellular components. Such interactions may induce DNA damage (Bilgin and Husunet 2025). These structural differences can affect not only the pharmacokinetics of the agents but also their potential to induce DNA damage and other biological effects.

The dose‐dependent relationship observed in the majority of studies suggests that the level of gadolinium exposure is crucial in determining its genotoxic effects. The potential mechanism behind this genetic damage is likely related to oxidative stress, as indicated by several studies (Cho et al. 2014; Donnelly et al. 2005). Gadolinium ions may induce the production of reactive oxygen species (ROS), which can cause DNA strand breaks, chromosome damage, and genomic instability. Furthermore, this oxidative stress may also impair DNA repair mechanisms, increasing the risk of long‐term genetic damage. These findings emphasize the importance of controlling gadolinium exposure during medical procedures to minimize potential genotoxic risks. The results showed that two studies identified that genotoxicity induced by gadolinium was caused by oxidative stress (Cho et al. 2014; Donnelly et al. 2005), whereas one study showed a decrease of DNA repair capacity after gadolinium exposure (Yongxing et al. 2000). It is well established that ROS are genotoxic agents either in vitro or in vivo (Vahidi et al. 2024). Additionally, it is important to stress that a total of five studies demonstrated that gadolinium was capable of inducing apoptosis, an important biological mechanism of cytotoxicity (Berthenloot et al. 2021; Cho et al. 2014). The induction of cytotoxicity, whether with or without DNA damage, has been previously demonstrated when exposed to certain chemical agents (Hartmann and Speit 1995). However, cytotoxicity is considered a relevant endpoint when conducting genotoxicity studies because cytotoxicity may induce DNA fragmentation by caspases, which could result in false‐positive outcomes (Tice et al. 2000).

Also, rather than gadolinium, other contrast agents could be used in MRI, such as common contrasts based on manganese and iron oxides (Gendron et al. 2024). In view of our findings, we believe that alternative elements should be studied in more depth so a comparison of damages assessment could be performed. This would be very helpful in selecting the least deleterious element for MRI tests, preserving patients' systemic health. By comparison, it has been established that patients with moderate or severe renal disease may be adversely affected by exposure to GBCAs. This exposure could potentially impair renal function through pro‐inflammatory and profibrotic mechanisms (Coimbra et al. 2024). Nonetheless, current data show that a single exposure to macrocyclic GBCAs seems to be safe in animals with normal kidney function, while long‐term toxicity associated with gadolinium retention warrants further investigation. This information is crucial, as the kidneys are the primary organs responsible for the excretion of gadolinium and GBCAs before they are removed from the body. Moreover, another review showed that the retention of gadolinium in the body persists even when renal disease is not present (Sharma et al. 2023). Lastly, a review published in 2023 highlights a shift in the understanding of GBCAs (Akbas et al. 2023). Contrary to previous beliefs that these agents were rapidly and completely eliminated from the human body following injection, emerging research indicates that gadolinium ions (Gd^3+^) can be retained in the body. This retention has been linked to competition with calcium ions (Ca^2+^), potentially disrupting calcium‐gated channels and interfering with various biological processes (Akbas et al. 2023). Such disruptions may lead to the formation of potentially toxic endpoints (Akbas et al. 2023). All in all, further investigation is needed to fully elucidate the mechanisms behind this toxicity.

In conclusion, this review provides evidence suggesting that gadolinium exposure may induce genotoxicity in mammalian cells, particularly under high‐dose or prolonged exposure scenarios. Given the potential risks, further research, especially in vivo studies, is urgently needed to clarify the long‐term effects of gadolinium retention in tissues. Additionally, the development and evaluation of alternative contrast agents should be prioritized to ensure safer imaging options for patients. Because exposure is performed in a short period of time in some specific cases for better MRI resolution, the use of gadolinium appears to be safe. However, we recommend that MRI scans with gadolinium contrast should be prescribed only when absolutely necessary. Finally, the implications of these findings underscore the need for improved safety protocols in workplaces where gadolinium is handled, ensuring better protection for workers involved in diagnostic imaging.

## Author Contributions

Study design: Thiago Guedes Pinto and Daniel Araki Ribeiro. Data search: Thiago Guedes Pinto and Daniel Araki Ribeiro. Data analysis: Thiago Guedes Pinto, Rogerio Aparecido Dedivits, and Daniel Araki Ribeiro. Writing the paper: Thiago Guedes Pinto, Rogerio Aparecido Dedivits, and Daniel Araki Ribeiro.

## Ethics Statement

The authors have nothing to report.

## Consent

The authors have nothing to report.

## Conflicts of Interest

The authors declare no conflicts of interest.

## Data Availability

Data sharing are not available to this article.
